# Supplementary material for: Relative model selection of evolutionary substitution models can be sensitive to multiple sequence alignment uncertainty
Source: BMC Ecol Evol. 2021 Nov 29;21:214. doi: 10.1186/s12862-021-01931-5 (PMC8628390; doi:10.1186/s12862-021-01931-5)
Supplement: Supplementary file 1 — Additional file 1: Additional Figures and Tables. [file 12862_2021_1931_MOESM1_ESM.pdf]

Supplementary Information: *Relative model selection of evolutionary substitution models is sensitive to multiple sequence alignment uncertainty*

Stephanie J. Spielman* and Molly Miraglia

*Corresponding author: spielman@rowan.edu (mailto:spielman@rowan.edu)

Additional Figures

Figure S1

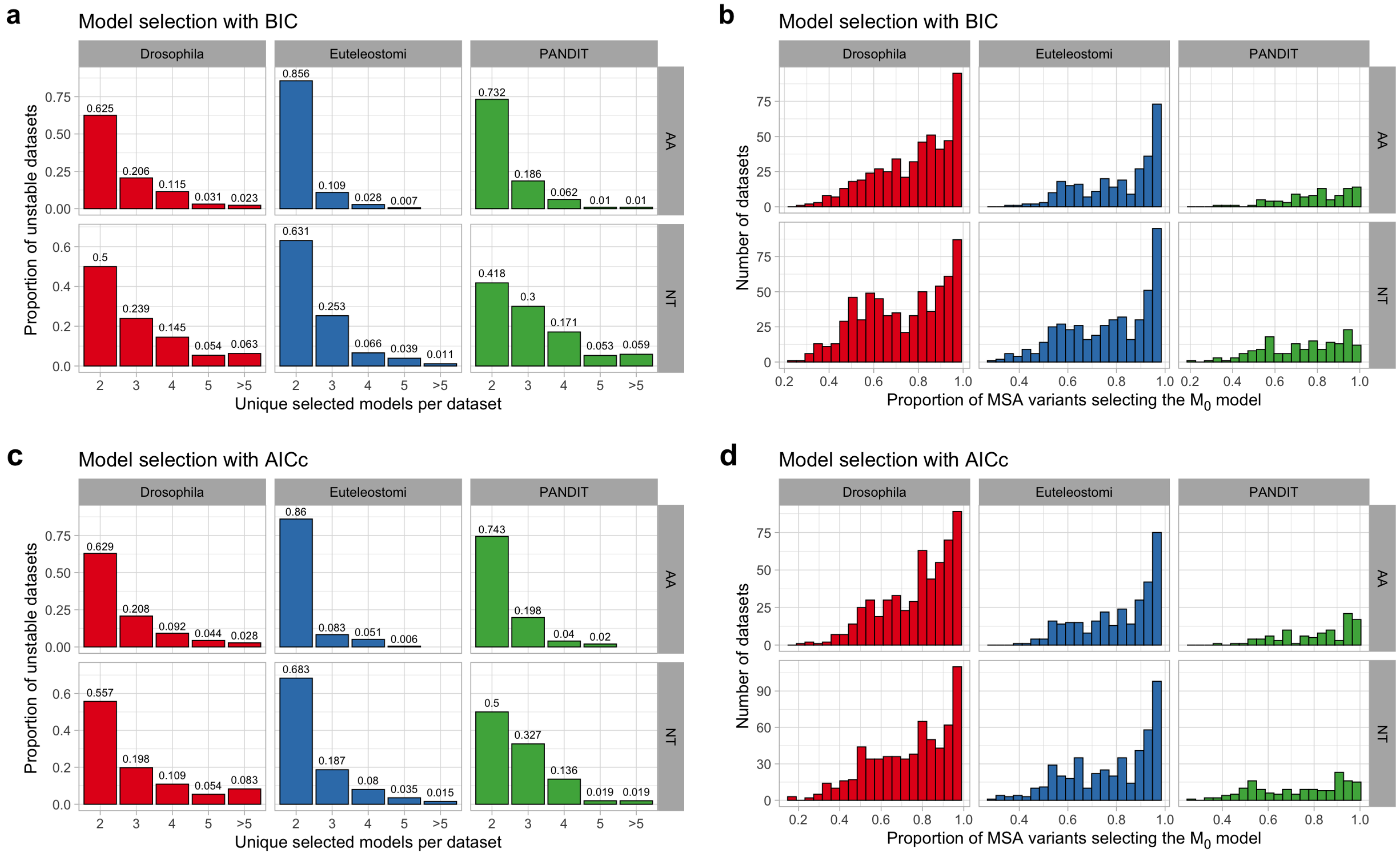

Figure S2

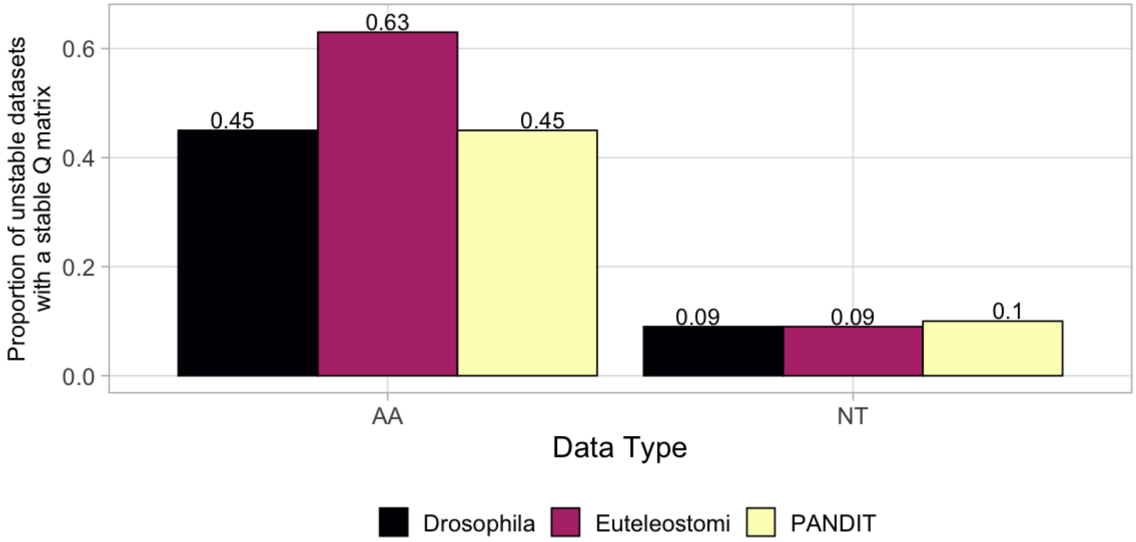

Figure S3

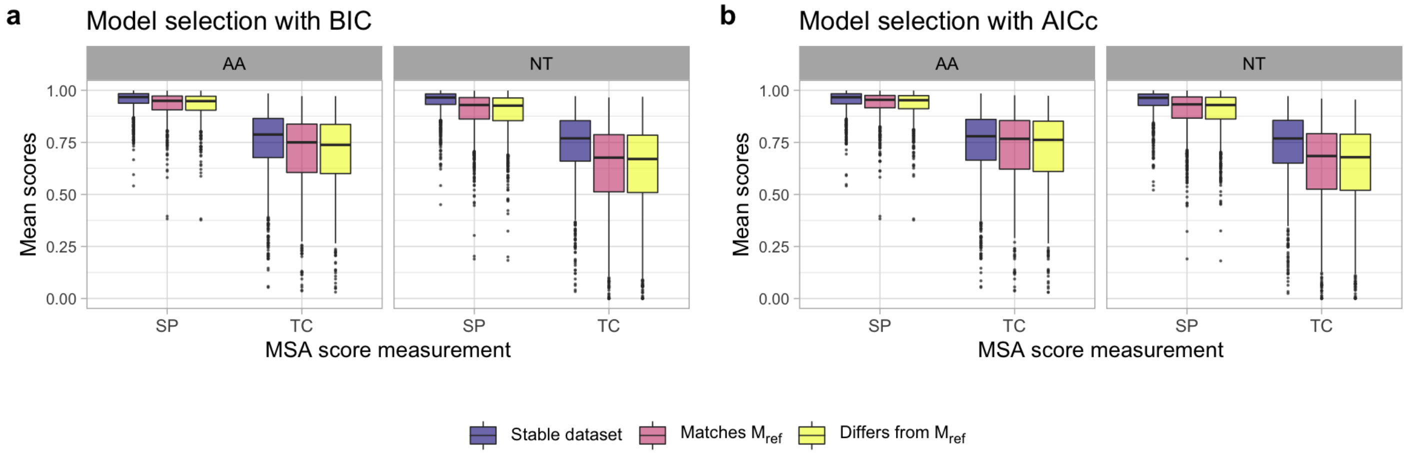

Additional Tables

Table S1

SP and TC scores comparisons among groups

Model selection with BIC

Datatype	Score type	Comparison	Effect size (99% CI)	Adjusted P-value
AA	SP	differs-matches	-0.002 (-0.011, 0.008)	0.867
**AA**	**SP**	**stable-matches**	**0.023 (0.015, 0.032)**	**0.000**
**AA**	**SP**	**stable-differs**	**0.025 (0.017, 0.033)**	**0.000**
AA	TC	differs-matches	-0.006 (-0.034, 0.021)	0.769
**AA**	**TC**	**stable-matches**	**0.049 (0.026, 0.073)**	**0.000**
**AA**	**TC**	**stable-differs**	**0.056 (0.032, 0.079)**	**0.000**
NT	SP	differs-matches	-0.003 (-0.015, 0.009)	0.739
**NT**	**SP**	**stable-matches**	**0.05 (0.038, 0.062)**	**0.000**
**NT**	**SP**	**stable-differs**	**0.053 (0.041, 0.065)**	**0.000**
NT	TC	differs-matches	-0.007 (-0.033, 0.019)	0.699
**NT**	**TC**	**stable-matches**	**0.105 (0.079, 0.132)**	**0.000**
**NT**	**TC**	**stable-differs**	**0.112 (0.086, 0.139)**	**0.000**

Table S2

SP and TC scores comparisons among groups

Model selection with AICc

Datatype	Score type	Comparison	Effect size (99% CI)	Adjusted P-value
AA	SP	differs-matches	-0.002 (-0.012, 0.007)	0.762
**AA**	**SP**	**stable-matches**	**0.016 (0.008, 0.025)**	**0.000**
**AA**	**SP**	**stable-differs**	**0.019 (0.01, 0.027)**	**0.000**
AA	TC	differs-matches	-0.008 (-0.034, 0.018)	0.653
**AA**	**TC**	**stable-matches**	**0.025 (0.001, 0.048)**	**0.006**
**AA**	**TC**	**stable-differs**	**0.033 (0.009, 0.056)**	**0.000**
NT	SP	differs-matches	-0.002 (-0.014, 0.01)	0.831
**NT**	**SP**	**stable-matches**	**0.044 (0.032, 0.056)**	**0.000**
**NT**	**SP**	**stable-differs**	**0.046 (0.034, 0.058)**	**0.000**
NT	TC	differs-matches	-0.006 (-0.032, 0.02)	0.788
**NT**	**TC**	**stable-matches**	**0.095 (0.068, 0.121)**	**0.000**
**NT**	**TC**	**stable-differs**	**0.101 (0.074, 0.127)**	**0.000**
